# A proposal for coping strategies on burnout among Japanese resident physicians

**DOI:** 10.1002/jgf2.662

**Published:** 2023-12-26

**Authors:** Kosuke Ishizuka, Kiyoshi Shikino, Akira Kuriyama, Yoshito Nishimura, Emiri Tanaka, Saori Nonaka, Michito Sadohara, Mitsuru Moriya, Noriko Yamamoto, Yohnosuke Wada, Tetsuya Makiishi

**Affiliations:** ^1^ Department of General Medicine Yokohama City University School of Medicine Yokohama Japan; ^2^ Department of Community‐oriented Medical Education Chiba University Graduate School of Medicine Chiba Japan; ^3^ Department of General Medicine Chiba University Hospital Chiba Japan; ^4^ Department of Primary Care & Emergency Medicine Kyoto University Hospital Kyoto Japan; ^5^ Department of Medicine, John A. Burns School of Medicine University of Hawai'i Honolulu Hawaii USA; ^6^ The Jikei University School of Medicine Minato‐ku Japan; ^7^ Department of General Medicine Taito Hospital Taito‐ku Japan; ^8^ Department of Community, Family, and General Medicine Kumamoto University Hospital Kumamoto Japan; ^9^ Department of Psychosomatic Internal Medicine Health Sciences University of Hokkaido Ishikari‐gun Japan; ^10^ Yamamoto Clinic Yokohama‐City Japan; ^11^ Department of Cardiovascular Surgery Jichi Medical University Saitama Medical Center Saitama Japan; ^12^ Department of General Medicine Shimane University Hospital Izumo Japan

To the Editor,

Burnout is a syndrome conceptualized by emotional exhaustion, depersonalization, and a diminished sense of personal achievement.^1^, ^2^ Physician burnout has several negative effects, including medical errors and mental ill health.^2^, ^3^ It is worth addressing that the prevalence of burnout among resident physicians in Japan is high at approximately 30%.^4^ Herein, we, the members of the American College of Physicians Japan Chapter Physicians' Well‐being Committee, report factors contributing to burnout of Japanese resident physicians and propose specific countermeasures (Table 1). Our recommendations are the result of focus group discussions with individuals of broad expertise and recent evidence, ensuring that they are grounded and directly relevant to the current challenges faced by resident physicians in Japan.

**TABLE 1:** Factors contributing to burnout and propose specific countermeasures among resident physicians.

Causes of burnout	Specific countermeasures against burnout
Communication problems in the workplace	Mentoring Sharing of policies within the practice team Change of practice team Meta‐awareness of one's feelings Analysis and reflection on difficult patient factors Developing empathy
2 Maladjustment to the job	Measures in line with reforms in the way doctors work in Japan Limitation or reduction of work Introduction of night flow Mandatory rest after duty shift Setting incremental goals through coaching
3 Multiple transfers and environmental changes	Assess the current state of knowledge, skills, and needs of resident physicians Limit the number of patients to be assigned at the beginning of the transfer Moderate expectations and acknowledge individual differences in adaptability
4 Low job satisfaction	Coaching to set work goals Focus on parts of the job that provide autonomy and enjoyment through coaching Create a platform for proactive dialogue with role models of a similar generation

## COMMUNICATION PROBLEMS IN THE WORKPLACE

Poor communication and stress in relationships with medical staff and patients may contribute to burnout among resident physicians.^2^, ^3^ Because most physicians in Japan start their careers without previous work experience, their communication skills with medical staff and patients may be underdeveloped. Mentoring, sharing plans within the medical team, and changing the teams may improve Communication with other medical staff. Resident physicians should also learn skills for coping with difficult patients who display strong negative feelings toward the physician.^5^ To cope with difficult patient encounters, metacognition of their own feelings, analysis on factors of difficult situations, and improvement in the capacity to empathize are important.^5^

## MALADJUSTMENT TO THE JOB

Long working hours, increased workload, sleep deprivation owing to duty shifts, and increased burden of COVID‐19 treatment are also risk factors for burnout.^2^, ^3^ Measures in line with the work reform of physicians in Japan, such as limiting or reducing work, introducing night flow, and mandatory rest after shifts, may be effective. Resilience can be improved by addressing “motivation” through coaching, setting incremental goals, building on successes to increase confidence, and setting new goals.^1^

## MULTIPLE TRANSFERS AND ENVIRONMENTAL CHANGES

In Japan, factors contributing to burnout among resident physicians include rotations through multiple departments and affiliated hospitals and changes in the environment, including community medicine and “*tasuki‐gake*” training (clinical training in which resident physicians work alternately between primary hospitals and external hospitals/clinics on a 1 year basis). It may be important to limit the number of patients to be assigned at the beginning of the rotation and to simultaneously assess the resident physician's performance. In addition, although changes in the environment increase the number of tasks to be acquired, it is important to modify one's mindset, for example, to recognize that the speed of acquiring new skills varies among individuals and to regard environmental changes as steps in career development.^1^, ^3^

## LOW JOB SATISFACTION

Among resident physicians, a lack of meaning in work, a lack of job autonomy, and a sense of helplessness contribute to burnout. To address low job satisfaction, it is important to focus, through coaching, on parts of the job that provide autonomy and enjoyment.^1^, ^3^ Furthermore, it is important to create a platform that enables clinical residents to actively interact with role models of a close generation to address low job satisfaction.

Identifying causes of burnout and providing specific countermeasures could help prevent burnout among resident physicians. Additional problems related to burnout among resident physicians should be identified, and interventions to reduce their burnout need to be sought.

## AUTHOR CONTRIBUTIONS

All authors had access to the data and a role in writing the manuscript.

## FUNDING INFORMATION

None.

## CONFLICT OF INTEREST STATEMENT

None.
